# *ACE2* deficiency exacerbates obesity-related glomerulopathy through its role in regulating lipid metabolism

**DOI:** 10.1038/s41420-022-01191-2

**Published:** 2022-09-30

**Authors:** Yin-Yin Chen, Han Hong, Yu-Ting Lei, Jia Zou, Yi-Ya Yang, Li-Yu He

**Affiliations:** 1 Department of Nephrology, Hunan Provincial People’s Hospital, The First Affiliated Hospital of Hunan Normal University, Changsha Clinical Research Center for Kidney Disease, Hunan Clinical Research Center for Chronic Kidney Disease, Changsha, 410000 Hunan Province P. R. China; 2 Department of Nephrology, The Second Xiangya Hospital of Central South University, Hunan Key Laboratory of Kidney Disease and Blood Purification, Changsha, 410011 Hunan Province P. R. China

**Keywords:** Mechanisms of disease, Glomerular diseases

## Abstract

Obesity-related glomerulopathy is a secondary glomerular disease and its incidence has been increased globally in parallel with the obesity epidemic. ORG emerged as a growing cause of end-stage renal disease in recent years. Unbalanced production of adipokines at the adipose tissue as well as low-grade inflammatory processes play central roles in ORG progression. ORG mouse model with ACE2-knockout was generated and kidney injury was evaluated by biochemistry and histological staining assays. Protein and mRNA expressions were quantified by ELISA, western blot or qRT-PCR methods. ACE2 deficiency aggravated ORG-related renal injuries and stimulated both lipid accumulation and inflammatory responses. Further, Nrf2 pathway was deactivated upon ACE2-knockout. By contrast, ACE2 overexpression reactivated Nrf2 pathway and ameliorated ORG symptoms by decreasing fat deposition and reducing inflammatory responses. Our data demonstrated that ACE2 exerted the beneficial effects by acting through Nrf2 signaling pathway, suggesting the protective role of ACE2 against lipid accumulation and inflammatory responses in ORG pathogenesis.

## Introduction

Obesity-related glomerulopathy (ORG) is a type of chronic kidney disease (CKD) that is pathologically associated with overweight and metabolic syndrome [1]. In parallel with the growing obesity rates across the world, the prevalence of ORG has significantly increased during the last decades [2]. Education about the risks of overweight and advocating for healthy lifestyle have been demonstrated as effective intervention for ORG at an early stage [3]. However, our understanding of ORG pathophysiology is rather limited and there exists no efficient therapeutic approach for aggravated course. In recent years ORG emerged as a growing cause of end-stage renal disease (ESRD) [4].

Obesity conveys fat disturbances [5]. Altered lipid metabolism and renal ectopic lipid accumulation play a central role in ORG progression. Lipid moieties directly induced lipo-toxicity in podocytes, renal proximal tubular cells, and mesangial cells [6–8]. Hypoadiponectinemia causes intraglomerular injuries in type 2 diabetic patients [9] and leads to increased glomerular filtration rate (GFR) as well as elevated albuminuria [10]. In addition, visceral fat triggers adipose inflammatory responses by stimulating the expression of proinflammatory cytokines, including IL-1β, TNF-α, and IL-6 [11, 12]. The unbalanced production of adipokines in the adipose tissue, is the mechanism of the low-grade inflammatory processes in patients with obesity or ORG [13, 14]. The chronic inflammatory state further promotes cardiovascular disease and contributes to the rising mortality in ESRD patients [15]. Questions regarding the regulation network of lipid molecules and proinflammatory cytokines in ORG pathophysiology remain to be thoroughly elucidated.

Angiotensin-converting enzyme 2 (ACE2) is a membrane-bound carboxypeptidase and hydrolyzes angiotensin peptides. ACE2 was reported to ameliorate diabetic nephropathy by attenuating the renal fibrosis [16, 17]. Although the pathogenesis of ORG is complex and poorly understood, we reason that it may share certain common mechanisms with diabetic nephropathy (DN). We speculate that ACE2 might be implicated in ORG onset. Studies demonstrated that ACE2 mediated liver lipid metabolism and reduced hepatic fat accumulation [18, 19]. Meanwhile, ACE2 deficiency was discovered to cause excess fat deposition in skeletal muscle [20]. However, it is unclear whether and how ACE2 affected the dysregulated lipogenesis in severe ORG complications.

Nuclear factor erythroid 2-related factor 2 (Nrf2) is a basic-leucine zipper (bZip) protein that controls the cellular toxicity and antioxidant defense by regulating a vast range of antioxidant genes [21]. The protective functions of Nrf2 against neurological damage [22], inflammatory and metabolic stresses have been extensively studied under physiological conditions [23, 24]. Our previous studies demonstrated that Nrf2 was inhibited in obesity-related nephropathy (ORN) and its degradation aggravated renal lipid deposition [25, 26]. Interestingly, Ma et al. reported that ACE2 overexpression induced Nrf2 upregulation in rostral ventrolateral medulla (RVLM) and suggested the potential role of Nrf2 in the antioxidant effect of ACE2 [27]. The interaction between ACE2 and Nrf2 pathway in ORG development remains to be studied.

In this study, we report that kidney injury and renal ectopic lipid accumulation were significantly exacerbated by ACE2 deficiency in ORG animal model. We also found that lipid droplet formation and expression of proinflammatory cytokines were enhanced by ACE2 knockdown in mouse renal PTCs. Further, we provide the first evidence that overexpression of ACE2 efficiently attenuated lipid deposition and inflammatory responses by acting through Nrf2 signaling pathway. Our results uncovered the protective role of ACE2 in ORG both animal and cellular models, and suggested its plausible application as a novel therapeutic target for ORG.

## Results

### Loss of ACE2 aggravated renal injuries in ORG mice

**Fig. 1:**
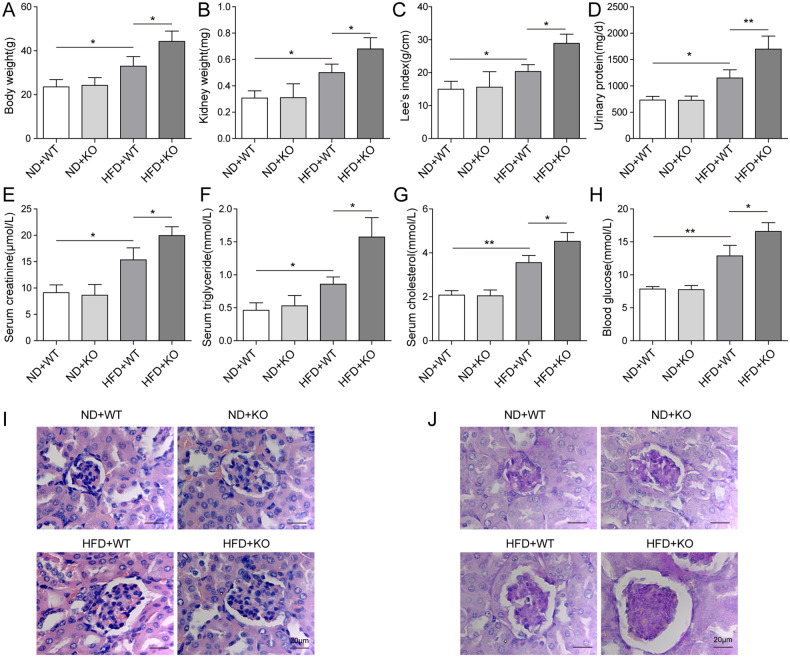
Loss of ACE2 aggravated renal injuries in ORG mice. WT and ACE-KO mice were fed with ND or HFD, and ORG animal model was generated by dividing the mice into four groups: ND + WT, ND + KO, HFD + WT, and HFD + KO. **A** Mouse body weight was recorded every three days. **B** Kidneys were dissected and weight was recorded after mice were euthanized. **C** Mouse body fat was calculated and HFD + ACE2-KO group displayed higher Lee’s index than the HFD + WT group. **D** HFD + ACE-KO mice showed 30% higher urinary protein concentration than HFD + WT group. Mouse blood was collected and **E** serum creatine concentration, **F** serum triglyceride concentration, **G** serum cholesterol concentration, as well as **H** blood glucose concentration were compared among the indicated groups above. **I** Renal damage in mouse specimens was assessed by H&E staining and severest damages were observed in HFD + ACE2-KO group, followed by HFD + WT group. Little or no damages were observed in ND + WT and ND + ACE2-KO groups. **J** PAS staining was conducted to confirm the histological changes. Data were representative images or were expressed as the mean ± SD of *n* = 3 experiments. *P < 0.05, **P < 0.01.

Previous studies demonstrated that ACE2 was implicated in type 2 diabetes mellitus (T2DM)-associated complications [28, 29]. In order to examine the potential function of ACE2 in ORG, we generated ORG animal model by feeding ACE2-deficient mice (ACE2-KO) and wild-type mice (WT) with HFD or ND for twelve weeks. As noted in Fig. 1A, HFD treatment induced a significant body weight gain in both ACE2-KO and WT mice. Intriguingly, ACE2-KO-HFD mice exhibited higher weight gain and heavier kidney than WT-HFD mice (Fig. 1B). However, we compared kidney/body weight ratio between the two groups above and no significance. Mouse body fat was estimated by calculating Lee Obesity Index (g/cm) using the formula [body weight (g) × 1000/naso-anal length (cm)]^1/3^. ACE2-KO mice fed with HFD displayed higher Lee’s index than the WT-HFD group (Fig. 1C). Next, we analyzed mouse urine samples and observed that the urinary protein concentration in ACE2-KO mice fed with HFD was 30% greater than in WT-HFD group (Fig. 1D). To verify whether the kidney function was altered by ACE2 deficiency, we collected blood from mice in each of the four indicated groups above and measured the serum creatinine. As shown in Fig. 1E, a clearly increased serum creatinine level was observed in ACE2-KO mice that were fed with HFD as compared with WT-HFD group. However, there was no significant difference in serum creatinine concentration between ACE2-KO and WT groups that were fed with ND. Concomitantly, we found that the serum triglyceride (Fig. 1F), serum cholesterol (Fig. 1G) and blood glucose (Fig. 1H) levels were largely upregulated in ACE2-KO-HFD mice in comparison to WT-HFD group. In contrast, these specific blood values remained almost unchanged between ACE2-KO mice and WT mice when they were both fed with NF.

To further validate the role of ACE2-deficiency in ORG-associated kidney damage, we isolated the renal tissues from mice in each group and performed histological assessments. As demonstrated in our H&E staining results (Fig. 1I), more severe glomerulosclerosis occurred in ACE2-KO mice fed with HFD than in WT-HFD group. In consistence, an independent PAS staining experiment revealed similar results (Fig. 1J). Taken together, our data corroborated that loss of ACE2 is correlated with ORG-associated renal injuries, and the inflammation reactions were exacerbated in ACE2-deficient ORG mice.

### Excess lipid was accumulated in renal tissues from ACE2-deficient ORG mice

**Fig. 2:**
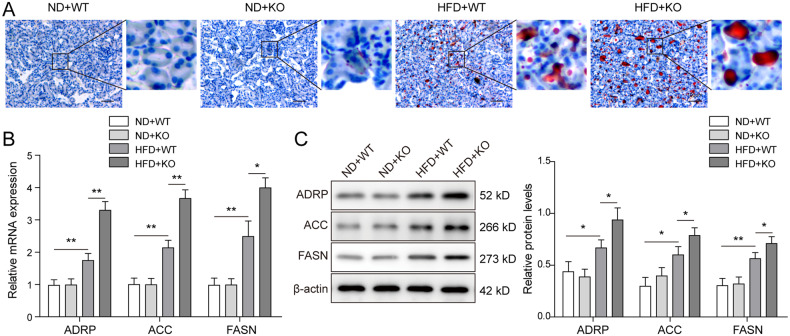
ACE2 deficiency induced excess fat deposition in ORG-mouse kidney tissues. **A** Lipid deposition in renal tissues was assessed by Oil Red O staining and HFD + ACE2-KO mice accumulated most fat. **B** qRT-PCR assay was performed to detect the relative mRNA expression of adipogenesis-related proteins. Highest expression of ADRP, ACC, and FASN was observed in HFD + ACE2-KO mice, followed by HFD + WT, ND + ACE2-KO and ND + WT mice. **C** The protein expression of the adipogenesis-related proteins was examined by western blot. Data were representative images or were expressed as the mean ± SD of *n* = 3 experiments. *P < 0.05, **P < 0.01.

Obesity often caused adipokines and ectopic lipid abnormalities, and ORG patients were often diagnosed with excessive lipid deposition in kidneys [30]. To investigate the mechanism through which the ORG-associated renal damages were affected by ACE2 deficiency, we dissected renal tissues from ORG mice and evaluated lipid accumulation by performing Oil Red O staining. As illustrated in Fig. 2A, the challenge with HFD, but not ND, stimulated lipid droplet formation in both ACE2-KO and WT mice, whereas the loss of ACE2 further enhanced lipid vacuole accumulation in mouse renal tissue. Next, we compared the expression of adipogenesis-related proteins in mouse kidney tissue from the ND + WT, ND + KO, HFD + WT, and HFD + KO groups. It could be noted from our qRT-PCR experimental results that relative mRNA expression of ADRP, ACC, and FASN in WT mice was upregulated upon HFD treatment (Fig. 2B), and further induced markedly in ACE2-KO mice. Consistently, western blot analysis revealed similar results for the protein expression of ADRP, ACC, and FASN (Fig. 2C). These results revealed the increased fat accumulation as well as the upregulated expression of the adipogenesis-related protein in ACE2-deficient ORG mice.

### Nrf2 signaling pathway was inhibited in ACE2-deficient ORG mice

**Fig. 3:**
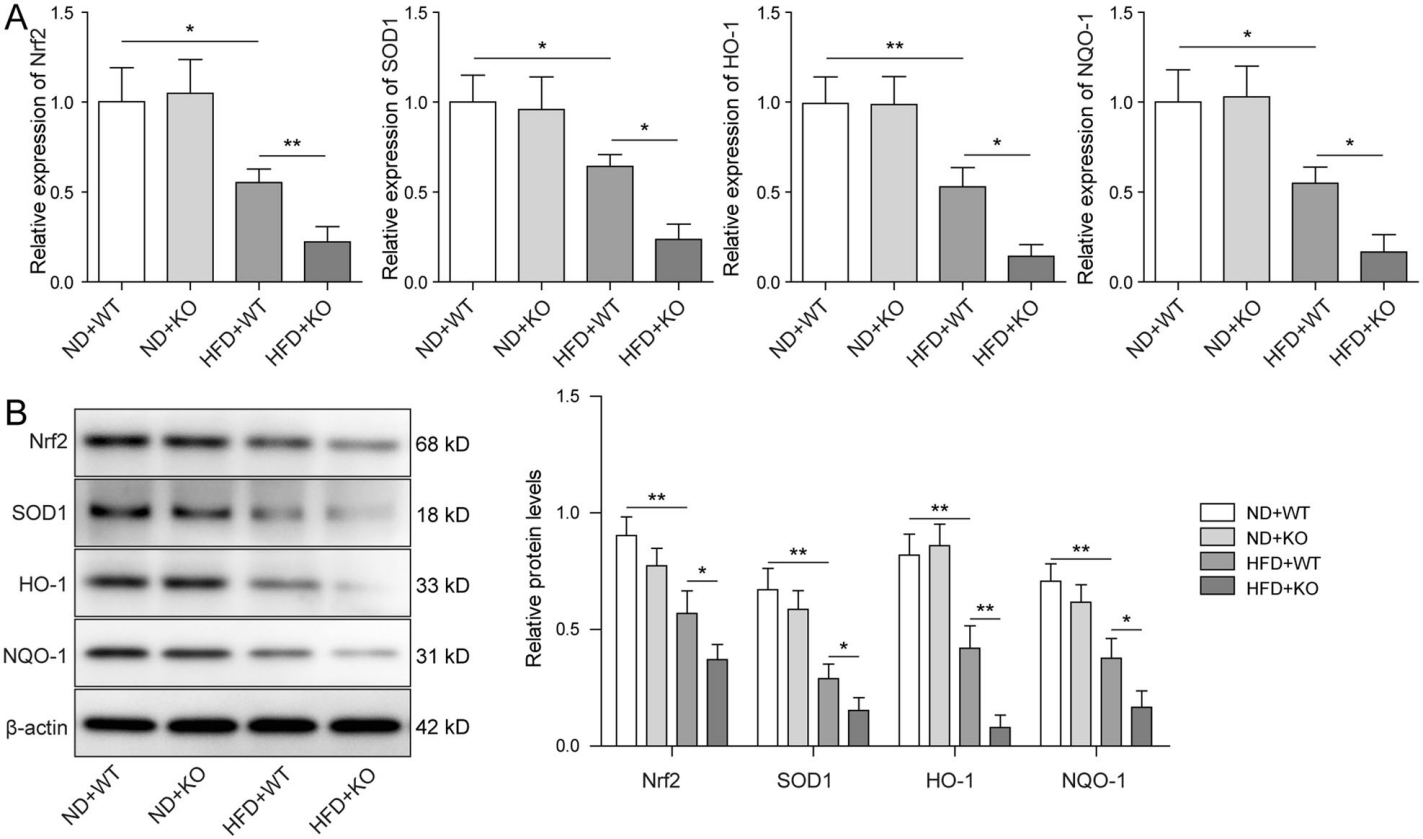
Nrf2 signaling pathway was inhibited in ACE2-deficient ORG mice. **A**, **B** The mRNA and protein expression levels of the Nrf2 pathway elements including Nrf2, SOD1, HO-1, NQO-1 were determined by qRT-PCR and western blot, respectively. The expression of Nrf2 and Nrf2 pathway effectors was decreased by HFD treatment and further eliminated in HFD + ACE2-KO mice. Data were representative images or were expressed as the mean ± SD of *n* = 3 experiments. *P < 0.05, **P < 0.01.

Nrf2 antioxidant signaling pathway is known to be linked with the aberrant activation of inflammasome in various chronic diseases [31]. Chen et al. recently reported that renal lipid deposition in ORG mice was accelerated by the silencing of Nrf2 [32]. Since we have, in accordance, detected enhanced expression of inflammatory cytokines in ACE2-deficient ORG mice, we addressed the question of whether Nrf2 pathway was altered by the ACE2 knockout. As shown in Fig. 3A, B, no significant difference was observed when the WT and ACE2-KO mice were fed with ND. On the contrary, the HFD challenge markedly reduced the expression level of the Nrf2 pathway components including Nrf2, SOD1, HO-1, NQO-1 in both WT and ACE2-KO mice. Notably, knockout of ACE2 almost totally abolished the expression of Nrf2 pathway elements. Our results together provided a solid substantiation that Nrf2 signaling pathway is deactivated in ORG animal model that carried the ACE2 knockout.

### Lipid deposition and inflammatory responses were induced by ACE2 knockdown in mouse renal PTCs

**Fig. 4:**
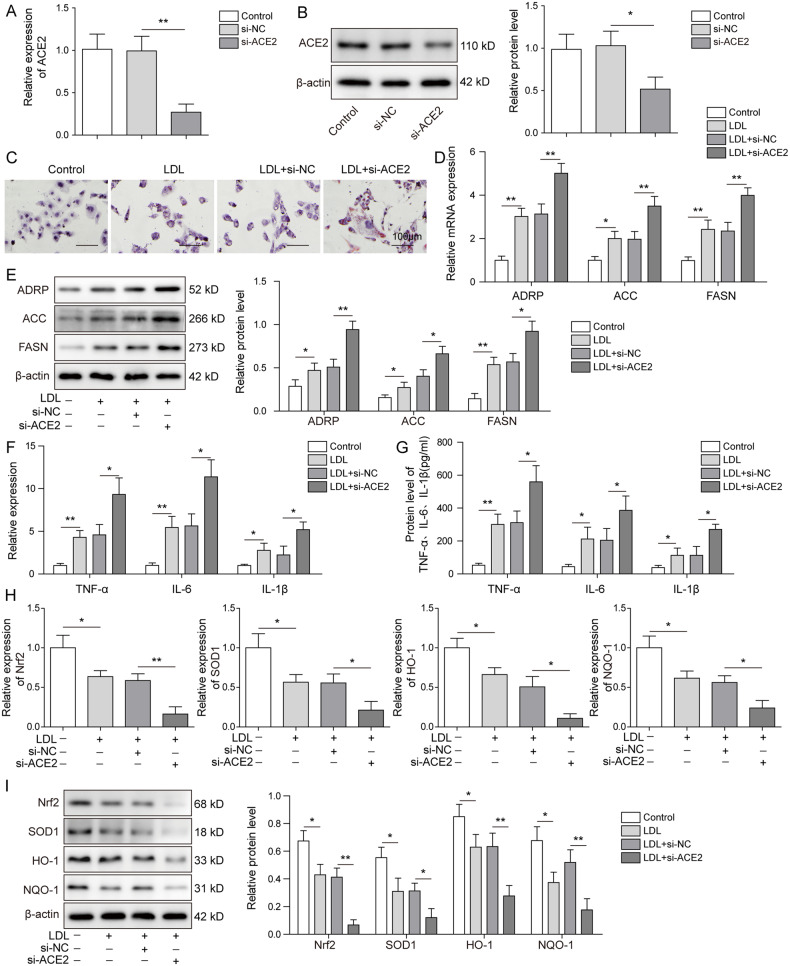
Lipid deposition and inflammatory responses were induced by ACE2 knockdown in mouse renal PTCs. Primary mouse PTCs were generated and lipo-toxicity was introduced by treating the PTCs with ox-LDL. ACE2 expression was silenced by transfecting ox-LDL-treated PTCs with si-ACE2, and transfection efficiency was evaluated by quantifying the ACE2 mRNA (**A**) and protein (**B**) expression. **C** Cellular lipid droplet formation was visualized by Oil Red O staining. ox-LDL-induced fat droplet formation was further stimulated by si-ACE2 treatment. Cellular lipid synthesis was assessed by detecting the expression of ADRP, ACC and FASN at mRNA (**D**) and protein levels (**E**). Expression profiles of the inflammatory cytokines were measured at mRNA (**F**) and protein (**G)** levels in PTCs. Stable inhibition of ACE2 stimulated expression of TNF-α, IL-6 and IL-1β compared with the control groups. **H**, **I** qRT-PCR and western blot were employed to evaluate the Nrf2 pathway elements including Nrf2, SOD1, HO-1, NQO-1 in the indicated groups. Nrf2 pathway was deactivated by si-ACE2 treatment. Data were representative images or were expressed as the mean ± SD of *n* = 3 experiments. *P < 0.05, **P < 0.01.

After demonstrating aggravated kidney damages as well as elevated lipid accumulation in ACE2-deficient ORG mouse model, we then tested whether the observations above were relevant in in vitro system. We have first established ORG cellular model by inducing lipo-toxicity in primary mouse renal PTCs with ox-LDL. Following that, ACE2 expression was suppressed by transfecting the cells with si-ACE2. As shown in Fig. 4A, ACE2 mRNA was efficiently silenced in ox-LDL-treated PTCs upon si-ACE2-transfection in comparison to the controls. WB analysis results showed consistently that ACE2 protein expression was markedly inhibited by si-ACE2 (Fig. 4B). Thus, the transfection efficiency of si-ACE2 was experimentally validated. Next, we assessed the cellular lipid droplet formation by performing Oil Red O staining. It could be noted from Fig. 4C that ox-LDL treatment clearly induced lipid droplet formation in PTCs, whereas the ACE2 knockdown further triggered the lipid accumulation, as compared to the control cells. Concomitantly, mRNA and protein expression of the adipogenesis-related proteins ADRP, ACC and FASN in PTCs were greatly stimulated by ox-LDL (Fig. 4D, E). As anticipated, we observed significantly increased expression of ADRP, ACC, and FASN at both protein and mRNA levels when ACE2 was silenced.

In addition, we evaluated whether the in vitro inflammatory activities were affected by ACE2 knockdown. As presented in our qRT-PCR analysis results, relative mRNA expression of the inflammatory cytokines TNF-α, IL-6, and IL-1β was enhanced in PTCs upon ox-LDL treatment, and further boosted when ACE2 was silenced (Fig. 4F). Utilizing WB assay, we observed consistent results and demonstrated the promoting effect of si-ACE2 on TNF-α, IL-6 and IL-1β at protein level (Fig. 4G). Interestingly, ox-LDL treatment of PTCs markedly reduced the protein expression of the Nrf2 pathway components including Nrf2, SOD1, HO-1, whereas ACE2 knockdown completely abrogated the expression of the Nrf2 pathway elements (Fig. 4H, I). These findings showed that in vitro deletion of ACE2 played a vital role in cellular lipid deposition and greatly stimulated the inflammatory processes. Moreover, Nrf2 pathway, the bridging linker between inflammasome and ORG progression, was notably attenuated by ACE2 knockdown.

### ACE2 attenuated lipid deposition and inflammatory responses by acting through Nrf2 pathway

**Fig. 5:**
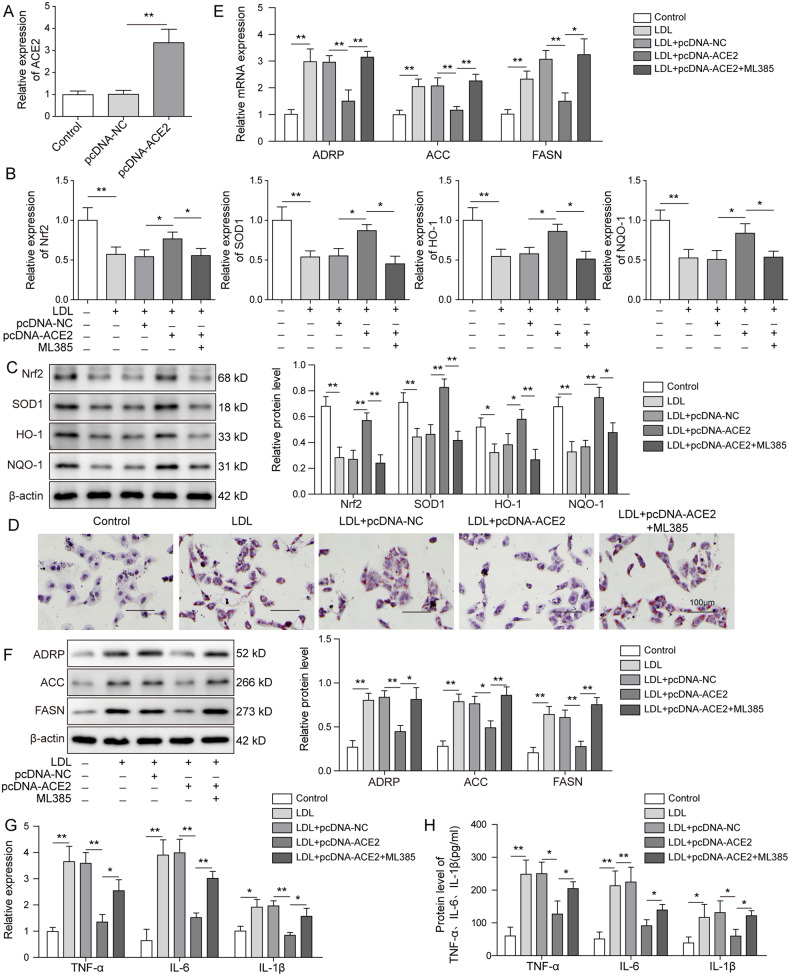
ACE2 attenuated lipid deposition and inflammatory responses by acting through Nrf2 pathway. Primary mouse renal PTCs were treated with ox-LDL and transfected with pcDNA-ACE2 for stable expression. **A** Transfection efficiency was calculated by qRT-PCR detection of the ACE2 mRNA expression. **B**, **C** qRT-PCR and western blot analysis were performed to measure the protein expression of Nrf2 signaling pathway elements including Nrf2, SOD1, HO-1, and NQO-1. ACE2 overexpression reactivated Nrf2 pathway, which was inhibited by ox-LDL, and the Nrf2-inhibitor ML385 abrogated the effect above. **D** Cellular lipid droplet formation was visualized by Oil Red O staining. Cellular lipid synthesis was evaluated by examining the expression of ADRP, ACC, and FASN at mRNA (**E**) and protein levels (**F**). Expression profiles of the inflammatory cytokines were determined at mRNA (**G**) and protein (**H**) levels. ACE-2 overexpression diminished expression of TNF-α, IL-6, and IL-1β, which was rescued by ML385. Data were representative images or were expressed as the mean ± SD of *n* = 3 experiments. *P < 0.05, **P < 0.01.

To address the question of whether Nrf2 participated in the inflammatory reactions mediated by ACE2, we first transfected the ox-LDL-treated mouse renal PTCs using pcDNA-ACE2 for stable overexpression. qRT-PCR assay revealed a 4-fold increase of ACE2 mRNA expression upon pcDNA-ACE2 transfection (Fig. 5A). As shown in western blot analysis results, protein expression of the Nrf2 pathway components including Nrf2, SOD1, HO-1 was largely suppressed by the treatment with ox-LDL and conversely induced by ACE2 overexpression (Fig. 5B, C). In contrast, Nrf2 pathway was effectively deactivated by adding the Nrf2 inhibitor ML385 to the PTCs. Oil Red O staining indicated that ACE2 overexpression greatly attenuated lipid droplet formation in PTCs, which was stimulated by the ox-LDL-induced treatment (Fig. 5D). Moreover, mRNA expression of the adipogenesis-related proteins ADRP, ACC, and FASN, which was enhanced by ox-LDL, was notably reduced by ACE2-overexpression, suggesting a protective effect of ACE2 against ox-LDL-induced lipo-toxicity. However, the protective effect was abrogated by adding Nrf2 inhibitor ML385 to the cells (Fig. 5E). In consistence, we detected markedly lower protein expression of ADRP, ACC, and FASN in ACE2-overexpressing PTCs than in control cells (Fig. 5F). Next, we examined the impact of ACE2 on the cellular inflammation responses. As we speculated, the mRNA expression of the inflammatory cytokine TNF-α, IL-6, and IL-1β, which was induced by ox-LDL, was significantly inhibited by transfecting PTCs with ACE2 (Fig. 5G). Intriguingly, the anti-inflammatory effect of ACE2 was counteracted by adding Nrf2 inhibitor ML385 to the ACE2-overexpressing PTCs, and we detected reversely enhanced expression of the inflammatory cytokines at both mRNA and protein levels (Fig. 5H). These in vitro experimental results indicated that ACE2 overexpression indeed alleviated lipid deposition and inflammatory responses in PTCs, and the cellular protective effect of ACE2 was mediated through Nrf2 pathway.

## Discussion

The obesity rates have increased in last decades, and 13% of the world population were estimated to have a BMI > 30 [33]. In addition to its substantially growing prevalence, obesity represents a leading risk factor for various diseases such as diabetes, nonalcoholic fatty liver disease (NAFLD), cardiac complications, and ORG [34]. As a major cause of ESRD, ORG patients not only suffer from reduced life quality and social disadvantages like other obese patients, but also are exposed to rather high mortality rate and cardiovascular complications. It’s well accepted that nephrotic syndrome, including proteinuria and focal segmental glomerulosclerosis, was most likely caused by the massive or morbid obesity [2]. However, the pathogenesis of ORG is not well understood and there lacks an efficient therapeutic approach especially when it progresses to late stage. In the present study, we focused on the molecular function of ACE2 in the pathophysiology of ORG. We demonstrated that lipid accumulation and inflammatory responses were induced by ACE2 deficiency in our cellular and animal ORG models. Overexpression of ACE2 ameliorated lipid deposition and inflammatory responses by acting through Nrf2 signaling pathway.

ACE2 is a zinc-containing metalloenzyme that acts as the membrane chaperone for SLC6A19 and the major entry point receptor for different coronavirus types. Liu et al. showed recently that transduction of ACE2 attenuated glomerular fibrosis in modified mesenchymal stem cells (MSCs) and efficiently ameliorated diabetic nephropathy [35]. Given the fact that the pathogenesis of ORG and diabetic nephropathy involves similar genetic and cellular factors, it’s plausible that ACE2 also plays a role in ORG etiology. Indeed, we observed that ORG mice with ACE2-knockout gained body weight much more rapidly than the standard ORG mice, and exhibited higher concentrations of urinary protein, serum creatine concentration, triglyceride concentration, cholesterol concentration, as well as blood glucose. These elevated values indicated that ACE2 deficiency aggravated the kidney injuries in ORG mice. Further, our data showed that expression of proinflammatory cytokines was stimulated by ACE2 knockout, suggesting that the exacerbated kidney injuries might be attributed to the chronic low-grade inflammation. ACE2 was reported to participate in lipid deposition regulation and reduced hepatic fat accumulation [18] as well as intramuscular fat accumulation [20]. Thus far there has been no research work in the literature about ACE2’s function in ORG-related lipid accumulation. To this end, we applied in vitro and in vivo experimental approaches and provide the first evidence that ACE2 participated in renal lipid metabolism regulation during ORG progression. Further, overexpression of ACE2 efficiently prevented renal ectopic lipid accumulation.

A previous study reported that ACE2 overexpression decreased sympathetic nerve activity (SNA) and central oxidant stress through activating the Nrf2 pathway [27]. Interestingly, we have previously reported that Nrf2 was inhibited in obesity-related nephropathy (ORN) and its degradation aggravated renal lipid deposition [25, 26]. Cheng et al. also demonstrated the renal protective effect of the activated Akt2/GSK-3β/Fyn/Nrf2 pathway in type 1 diabetic (T1D) mice by lowering the ectopic lipid-deposition [23]. Thus, we aimed at investigating whether and how Nrf2 pathway was involved in ACE2 deficiency-induced ORG development. Our work showed that the ACE2-knockout in ORG mice deactivated the Nrf2 signaling pathway by inhibiting the expression of pathway components, including Nrf2, SOD1, HO-1, and NQO-1. By contrast, ACE2 overexpression reactivated Nrf2 pathway and improved ORG conditions by reversing both lipid accumulation and inflammatory responses in ox-LDL-treated renal PTCs. Nevertheless, our study didn’t investigate the possible upstream pathways that directly regulate the ACE2/Nrf2 axis. Further, our data doesn’t exclude other molecular networks which might concomitantly modulate the ORG progression with ACE2 and Nrf2 pathway. We would also need perform clinical research and verify the disease mechanisms.

Collectively, we showed that ACE2-knockout exacerbated ORG progression by aggravating lipid accumulation and activating inflammatory responses at both cellular and animal levels. In vitro overexpression of ACE2 largely reversed the adverse effects of ACE2 deficiency by modulating the Nrf2 pathway. Our work provides a novel insight into the mechanisms of ORG pathogenesis and may broaden the current perspective for ORG treatment.

## Materials and methods

### Isolation of primary renal proximal tubule cells (PTCs) and cell culture

The isolation of PTCs was performed as previously described with minor modifications [36]. Renal cortices were dissected from C57/BL6-mice (male, 21–30 days) and cut into 1 mm^3^ fragments in ice-cold dissection solution (DS) (1× HBSS with 2.5 mM HEPES pH 7.4, 35 mM glucose and 4 mM sodium bicarbonate). Next, the cortical tissue was digested by 0.1 U/mL collagenase (Sigma, St Louis, MO, USA) in DS at 37 °C. The suspension was centrifuged at 300 × *g* for 5 min and the cleared supernatant was passed sequentially through 250-µm and 80-µm nylon mesh sieves. The remaining long proximal tubule fragments were washed in 1× PBS, resuspended in DS containing 1% BSA (Sigma, St Louis, MO, USA) at 37 °C and finally maintained in Dulbecco’s Modified Eagle’s Medium/Nutrient Mixture F-12 (DMEM/F-12) supplemented with 10% fetal bovine serum (FBS, Gibco, Grand Island, NY, USA), 100 U/mL penicillin, 0.1 mg/mL streptomycin (Sigma, St Louis, MO, USA), 1% L-glutamine, 15 mM HEPES, 50 mM hydrocortisone, 5 µg/mL insulin, 5 µg/mL transferrin and 50 nM sodium selenite. Non-adherent cells were removed after 24 h by changing the culture medium.

For the in vitro oxidized low-density lipoprotein (oxLDL) treatment, native human LDL (Sigma, St Louis, MO, USA) was oxidized by 5 µM CuSO_4_ at 37 °C and dialysed in PBS containing 200 µM EDTA. After the oxidation level was confirmed by the thiobarbituric acid (TBA) test, 50 µg/mL oxLDL was added to confluent PTCs, which were pretreated with 20 µmol/L antioxidant probucol (Sigma, St Louis, MO, USA) or 250 µg/mL LOX-1 receptor inhibitor polyinosinic acid (Sigma, St Louis, MO, USA). After 24 h stimulation, oxLDL was removed from PTCs, cells were washed and cultured for additional 24 or 48 h before further analysis.

### Plasmid synthesis and cell transfection

Small interfering RNAs (siRNA) against ACE2 was generated by GenePharma (Shanghai, China) and further subcloned into pcDNA3.1 vector. ACE2 expression plasmid pcDNA-ACE2 was constructed by inserting ACE2-coding sequence to pcDNA3.1 vector. Freshly isolated mouse PTCs at passage 2–3 were plated on a 6-well plate at a seeding density of 2 × 10^5^ cells/well and grown to 50% confluency. For stable inhibition or expression of ACE2, cells were transfected with si-ACE2 or pcDNA-ACE2 using Lipofectamine2000 (Invitrogen). Empty vector pcDNA-NC was used as negative control. Cells were harvested 48 h after transfection and lysed for subsequent analysis.

### Total RNA extraction and quantitative real-time PCR (qRT-PCR)

Primary PTCs and kidney tissue samples were freshly snap frozen and RNA was extracted using Trizol Reagents (Invitrogen). The absorbance of isolated RNA purity was determined by UV spectroscopy at 260 and 280 nm respectively. Complementary DNA (cDNA) was synthesized by reverse transcribing 2 μg total RNA as template using a MyCycler Thermal Cycler (BioRad). qRT-PCR steps were conducted in a single tube using One Step SYBR PrimeScript RT-PCR kit (Takara, Dalian, China) on a 7900HT Fast Real-Time PCR System (Applied Biosystems) according to the product manuals. Raw data was analyzed using the 2^−ΔΔCt^ Ct method and relative expression of ACE2, ADRP, ACC, FASN, ACE2, TNF-α, IL-6 and IL-1β was normalized to the internal reference GAPDH. The qRT-PCR primers for ACE2, ADRP, ACC, FASN, ACE2, TNF-α, IL-6 and IL-1β were designed and synthesized by Sangon Biotech (Shanghai, China) Co., Ltd.

### Protein extraction and western blot

Total protein was extracted from freshly frozen primary PTCs and kidney tissue samples after being lysed in ice-cold RIPA lysis buffer containing 1× protease inhibitor cocktail (Sigma, St Louis, MO, USA). Protein concentration was determined using Bradford Protein Assay kit (Beyotime, China). Next, 40 μg of protein sample from the cleared lysates was loaded on a 12% sodium dodecyl sulfate polyacrylamide gel (SDS-PAGE) and subsequently electro-transferred to a nitrocellulose membrane. β-actin was used as a loading control. The membrane was incubated with 3% BSA for an hour at room temperature 1% BSA to block the non-specific bindings. Following the blocking step, primary antibodies against ADRP (Abcam, ab181452, 1:1000), ACC (Abcam, ab109368, 1:5000), FASN (Abcam, ab128870, 1:20,000 dilution), ACE2 (Abcam, ab108252, 1:3000 dilution), Nrf2 (Abcam, ab89443, 1:1000 dilution), SOD1 (Abcam, ab51254, 1:50,000 dilution), HO-1 (Abcam, ab52947, 1:2000 dilution), NQO1 (Abcam, ab34173, 1 µg/ml) and β-actin (Abcam, ab8226, 1 µg/ml) were diluted at the recommended concentrations and applied to the membrane for overnight incubation with agitation at 4 °C. On the next day, the blot was extensively washed with 100 µM PBST and incubated with diluted peroxidase-conjugated secondary antibody (Abcam, ab205718, 1:10,000) in blocking buffer for 1 hour at room temperature. Protein bands were developed by incubation with Pierce ECL substrates (Thermal Fisher, Rockford, USA) for 1 min and images were captured under Odyssey infrared scanner (Li-Cor Biosciences Inc.).

### Generation of obesity-related glomerulopathy (ORG) mice

Animal experimental protocols were approved by The Second Xiangya Hospital of Central South University and performed in compliance with relevant regulations. Twenty ACE2 wild-type (WT) and twenty ACE2 knockout (KO) C57/BL6 male mice (6-week old, 19–22 g) were obtained from Shanghai SLAC Laboratory Animal Co., Ltd and divided randomly into four groups: WT were fed with a normal diet (ND) (*n* = 10), WT were fed with a high-fat diet (HFD) (*n* = 10), KO were fed with a ND (*n* = 10), and KO were fed with a HFD (*n* = 10) for up to twelve weeks. ND containing fat accounting for 10% kcal was purchased from Beijing Huafukang Biological Technology Co. Ltd and HFD was purchased from Research Diets (Rodent Diet with 60% Kcal Fat, Research Diets, New Brunswick, USA). All animals were raised in an air-conditioned animal room at 25 °C with free access to food and clean water. Mouse body weight was recorded every three days. Mice were euthanized by decapitation after twelve weeks. Renal tissues were dissected and weighed. Half of the tissues were fixed in 4% PFA for histological analysis, and the other half was snap frozen in liquid nitrogen for qRT-PCR, ELISA, and western blot analysis.

### Hematoxylin and eosin (H&E) staining

The mouse kidney was rapidly dissected and exposed to ultrasound. Next, renal tissue was fixed with freshly prepared 4% paraformaldehyde for 12 h, embedded in paraffin wax and sliced into 4-µm thick coronal sections. After affixing to a microscope slide, paraffin was thoroughly dissolved away by two changes of the hydrocarbon solvent xylene. Before application of the hematoxylin nuclei stain, the slides were washed with 100% ethanol to remove xylene traces, followed by re-hydration with 95% and 70% ethanol. Hematoxylin was dissolved in the mordant aluminum ammonium sulfate and incubated with the slides for 10 min. The slides were rinsed in tap water and differentiated with 0.3% acid alcohol to remove the non-specific stain. Finally, non-nuclear elements were counter-stained with eosin for 3 min, and over-staining was washed away under tap water. Renal samples were dehydrated with increasing concentration of ethanol and several baths of xylene to remove excess water completely. A thin layer of Permount (VWR, South Plainfield, NJ, USA) was applied and the slides were imaged under a Leica microscope (DMIRB, Germany).

### Lipid accumulation analysis by Oil Red O staining

Oil Red O stock solution was prepared by dissolving 0.5 g Oil Red O (Sigma, St Louis, MO, USA) in 100 ml isopropanol and working solution was freshly prepared for each use by adding two parts of distilled water (dH_2_O) to three parts of stock solution in the fume hood. Mouse renal tissue was fixed in 4% buffered paraformaldehyde (PFA), pH 6.9 for an hour and frozen sections were cut at 4 µm. The slices were fixed to a microscope slide and fixative material was removed by briefly rinsing the slides with dH_2_O followed by 60% isopropanol. Prior to staining, isopropanol was gently removed from the tissue sections. The renal specimens were incubated with Oil Red O working solution for an hour, and then subjected to nuclear staining using 50 nM 4′,6-diamidino-2-phenylindole (DAPI).

To analyze the lipid droplet formation in cultured PTCs, cells were plated on a six-well plate and fixed with 4% PFA by 20 min incubation. After PFA was poured off and cells were washed twice with 1× PBS, PTCs were incubated briefly with 60% isopropanol and then with 2 ml Oil Red O working solution for 20 min. Oil Red O solution was discarded and counterstaining was performed by incubation with 50 nM DAPI for 3 min. Finally, DAPI solution was aspirated and cells were washed by dH_2_O with gentle rocking. Images were acquired under a Leica microscope.

### Periodic acid-Schiff (PAS) stain

Glycogen in mouse renal tissue was measured using PAS staining method. Briefly, renal tissue sections were fixed in 4% PFA and embedded in paraffin wax. The 5 µm paraffin section were thoroughly deparaffinized by incubating twice with xylene, each time 3 min. Once the slides were rehydrated with decreasing concentrations of ethanol, 0.5% (w/v) PAS (Sigma, St Louis, MO, USA) was applied to specimens and incubated for 15 min at room temperature. The oxidation process was terminated by washing the slides with dH_2_O and the aldehyde production was detected by immersing the slides in Schiff reagent (Sigma, St Louis, MO, USA) for 15 min. Counterstaining was performed by incubation with hematoxylin for 1 min, followed by rinsing under tap water. The slides were air-dried and examined under a Leica microscope.

### Enzyme-linked immunosorbent assay (ELISA)

PTCs were transfected with si-ACE2 or pcDNA-ACE2, treated with oxLDL and further maintained at 37° for 24 h. The expression levels of the inflammatory cytokines TNF-α, IL-6, and IL-1β were quantified using human TNF alpha ELISA kit (Abcam, UK, ab181421), human IL-6 ELISA kit (Abcam, UK, ab178013) and human IL-1 beta ELISA kit (Abcam, UK, ab197742) respectively by following the manufacturer’s protocol. Optical density (OD) of both samples and standards was measured at 450 nm on the Tecan reader.

### GFR (glomerular filtration rate) measurement

GFR was measured by calculating the clearance rate of sinistrin which was labeled with fluorescein isothiocyanate (FITC) (Fresenius-Kabi, Linz). Briefly, 5% FITC-sinistrin was administered through tail vein and blood was collected at 10, 30, 60, 90, 120, and 180 min after injection. Fluorescence readout at 485 nm excitation and 538 nm emission was recorded on a fluorometer.

### Serum creatinine determination

Serum creatinine levels were measured using the Creatinine Assay Kit (Abcam, ab65340) according to the manufacturer’s protocol. Briefly, the serum samples were first deproteinized by passing through a 10 kDa filter. 50 µL Creatinine reaction mix was prepared and incubated at 37 °C for an hour. Output at OD 570 nm was measured on a microplate reader.

### Statistical analysis

All experiments were performed at least three times. Data analysis was conducted using SPSS Statistics 20.0, and the data variation was measured by the standard deviation (SD). Significance in difference between two groups was compared using Student’s *t*-test. Differences among more than two independent groups were tested using one-way ANOVA (analysis of variance) and further specifically evaluated by Tukey’s post hoc test. A *P*-value smaller than 0.05 was considered as statistically significant for all analysis.

### Supplementary information

supplementary materials

Original Data File

Figure S1

Figure S2

Figure S3

## Data Availability

The datasets used and/or analyzed during the current study are available from the corresponding author on reasonable request.

## References

[1] D’Agati VD, Chagnac A, de Vries AP, Levi M, Porrini E, Herman-Edelstein M (2016). Obesity-related glomerulopathy: clinical and pathologic characteristics and pathogenesis. Nat Rev Nephrol.

[2] Kambham N, Markowitz GS, Valeri AM, Lin J, D’Agati VD (2001). Obesity-related glomerulopathy: an emerging epidemic. Kidney Int.

[3] O’Donoghue DJ, Stevens PE (2012). A decade after the KDOQI CKD/guidelines: a perspective from the United Kingdom. Am J Kidney Dis.

[4] Yang S, Cao C, Deng T, Zhou Z, Obesity-Related (2020). Glomerulopathy: a latent change in obesity requiring more attention. Kidney Blood Press Res.

[5] He L, Tang M, Xiao T, Liu H, Liu W, Li G (2018). Obesity-associated miR-199a/214 cluster inhibits adipose browning via PRDM16-PGC-1alpha transcriptional network. Diabetes..

[6] de Vries AP, Ruggenenti P, Ruan XZ, Praga M, Cruzado JM, Bajema IM (2014). Fatty kidney: emerging role of ectopic lipid in obesity-related renal disease. Lancet Diabetes Endocrinol.

[7] Kovesdy CP, Furth SL, Zoccali C (2017). Obesity and kidney disease: hidden consequences of the epidemic. Afr J Prim Health Care Fam Med.

[8] Xu T, Sheng Z, Yao L (2017). Obesity-related glomerulopathy: pathogenesis, pathologic, clinical characteristics and treatment. Front Med.

[9] Li H, Xiao Y, Liu H, Chen XY, Li XY, Tang WL (2011). Hypoadiponectinemia predicts impaired endothelium-independent vasodilation in newly diagnosed type 2 diabetic patients: an 8-year prospective study. Chin Med J.

[10] Eknoyan G (2011). Obesity and chronic kidney disease. Nefrologia.

[11] Hunley TE, Ma LJ, Kon V (2010). Scope and mechanisms of obesity-related renal disease. Curr Opin Nephrol Hypertens.

[12] Santini E, Lupi R, Baldi S, Madec S, Chimenti D, Ferrannini E (2008). Effects of different LDL particles on inflammatory molecules in human mesangial cells. Diabetologia..

[13] Ellulu MS, Patimah I, Khaza’ai H, Rahmat A, Abed Y (2017). Obesity and inflammation: the linking mechanism and the complications. Arch Med Sci.

[14] Teplan V, Vyhnanek F, Gurlich R, Haluzik M, Racek J, Vyhnankova I (2010). Increased proinflammatory cytokine production in adipose tissue of obese patients with chronic kidney disease. Wien Klin Wochenschr.

[15] Collins AJ, Foley RN, Chavers B, Gilbertson D, Herzog C, Johansen K (2012). ‘United States Renal Data System 2011 Annual Data Report: Atlas of chronic kidney disease & end-stage renal disease in the United States. Am J Kidney Dis.

[16] Batlle D, Wysocki J, Soler MJ, Ranganath K (2012). Angiotensin-converting enzyme 2: enhancing the degradation of angiotensin II as a potential therapy for diabetic nephropathy. Kidney Int.

[17] Tikellis C, Brown R, Head GA, Cooper ME, Thomas MC (2014). Angiotensin-converting enzyme 2 mediates hyperfiltration associated with diabetes. Am J Physiol Ren Physiol.

[18] Cao X, Yang F, Shi T, Yuan M, Xin Z, Xie R (2016). Angiotensin-converting enzyme 2/angiotensin-(1-7)/Mas axis activates Akt signaling to ameliorate hepatic steatosis. Sci Rep..

[19] Yang M, Ma X, Xuan X, Deng H, Chen Q, Yuan L (2020). Liraglutide attenuates non-alcoholic fatty liver disease in mice by regulating the local renin-angiotensin system. Front Pharm.

[20] Cao X, Lu XM, Tuo X, Liu JY, Zhang YC, Song LN (2019). Angiotensin-converting enzyme 2 regulates endoplasmic reticulum stress and mitochondrial function to preserve skeletal muscle lipid metabolism. Lipids Health Dis.

[21] Ma Q (2013). Role of nrf2 in oxidative stress and toxicity. Annu Rev Pharm Toxicol.

[22] Pajares M, Jimenez-Moreno N, Garcia-Yague AJ, Escoll M, de Ceballos ML, Van Leuven F (2016). Transcription factor NFE2L2/NRF2 is a regulator of macroautophagy genes. Autophagy..

[23] Cheng Y, Zhang X, Ma F, Sun W, Wang W, Yu J (2020). The role of Akt2 in the protective effect of fenofibrate against diabetic nephropathy. Int J Biol Sci.

[24] Rojo de la Vega M, Dodson M, Gross C, Mansour HM, Lantz RC, Chapman E (2016). Role of Nrf2 and autophagy in acute lung injury. Curr Pharm Rep..

[25] Chen Y, He L, Yang Y, Chen Y, Song Y, Lu X (2019). The inhibition of Nrf2 accelerates renal lipid deposition through suppressing the ACSL1 expression in obesity-related nephropathy. Ren Fail.

[26] Chen YY, Hong H, Lei YT, Zou J, Yang YY, He LY (2021). IkappaB kinase promotes Nrf2 ubiquitination and degradation by phosphorylating cylindromatosis, aggravating oxidative stress injury in obesity-related nephropathy. Mol Med.

[27] Ma A, Gao L, Wafi AM, Yu L, Rudebush T, Zhou W (2020). Overexpression of central ACE2 (angiotensin-converting enzyme 2) attenuates the pressor response to chronic central infusion of Ang II (Angiotensin II): a potential role for Nrf2 (nuclear factor [erythroid-derived 2]-like 2). Hypertension.

[28] Patel VB, Parajuli N, Oudit GY (2014). Role of angiotensin-converting enzyme 2 (ACE2) in diabetic cardiovascular complications. Clin Sci.

[29] Xie W, Wu D, Ren Y, Jiang Y, Zhang H, Yang S (2020). OIP5-AS1 attenuates microangiopathy in diabetic mouse by regulating miR-200b/ACE2. World Neurosurg.

[30] Mount P, Davies M, Choy SW, Cook N, Power D (2015). Obesity-related chronic kidney disease-the role of lipid metabolism. Metabolites..

[31] Ahmed SM, Luo L, Namani A, Wang XJ, Tang X (2017). Nrf2 signaling pathway: pivotal roles in inflammation. Biochim Biophys Acta Mol Basis Dis.

[32] Wang J, Jiang C, Zhang K, Lan X, Chen X, Zang W (2019). Melatonin receptor activation provides cerebral protection after traumatic brain injury by mitigating oxidative stress and inflammation via the Nrf2 signaling pathway. Free Radic Biol Med.

[33] Bluher M (2019). Obesity: global epidemiology and pathogenesis. Nat Rev Endocrinol.

[34] Ferguson EC, Stewart EK, Hannah C, Elder CJ (2020). Obesity: unrecognised or avoided? We are missing opportunities to ‘make every contact count’. Arch Dis Child.

[35] Liu Q, Lv S, Liu J, Liu S, Wang Y, Liu G (2020). Mesenchymal stem cells modified with angiotensin-converting enzyme 2 are superior for amelioration of glomerular fibrosis in diabetic nephropathy. Diabetes Res Clin Pr.

[36] Van der Hauwaert C, Savary G, Gnemmi V, Glowacki F, Pottier N, Bouillez A (2013). Isolation and characterization of a primary proximal tubular epithelial cell model from human kidney by CD10/CD13 double labeling. PLoS ONE.

