# Correction: *ACE2* deficiency exacerbates obesity-related glomerulopathy through its role in regulating lipid metabolism

**DOI:** 10.1038/s41420-023-01683-9

**Published:** 2023-10-23

**Authors:** Yin-Yin Chen, Han Hong, Yu-Ting Lei, Jia Zou, Yi-Ya Yang, Li-Yu He

**Affiliations:** 1 Department of Nephrology, Hunan Provincial People’s Hospital, The First Affiliated Hospital of Hunan Normal University, Changsha Clinical Research Center for Kidney Disease, Hunan Clinical Research Center for Chronic Kidney Disease, Changsha, 410000 Hunan Province P. R. China; 2 Department of Nephrology, The Second Xiangya Hospital of Central South University, Hunan Key Laboratory of Kidney Disease and Blood Purification, Changsha, 410011 Hunan Province P. R. China

**Keywords:** Mechanisms of disease, Glomerular diseases

Correction to: *Cell Death Discovery* 10.1038/s41420-022-01191-2, published online 30 September 2022

In this article, the first author name has been misspelled.

Yin-Yin Che should be read Yin-Yin Chen.

The original article has been corrected.

