# Supplementary material for: ACE2 deficiency exacerbates obesity-related glomerulopathy through its role in regulating lipid metabolism
Source: Cell Death Discov. 2022 Sep 30;8:401. doi: 10.1038/s41420-022-01191-2 (PMC9523180; doi:10.1038/s41420-022-01191-2)
Supplement: Supplementary file 1 — supplementary materials [file 41420_2022_1191_MOESM1_ESM.docx]

**FIG S1**. Protein expression of Laminin, Collagen IV, Fibronectin was examined by WB. Highest expression was observed in HFD+KO group, followed by HFD+WT, HD+KO and ND+WT groups. Data were representative images or were expressed as the mean ± SD of *n = 3* experiments. *, P < 0.05, **, P < 0.01.

**FIG S2.** Protein expression of ACE2 was examined in the frozen samples of kidney, lung and large intestine tissues. ACE2 expression in ACE2 KO mice was clearly higher than in WT mice. Data were representative images or were expressed as the mean ± SD of *n = 3* experiments. *, P < 0.05, **, P < 0.01.

**FIG S3.** Protein expression of TNFα, IL-6, IL-1β in kidney tissue was measured by ELISA. Highest expression was observed in HFD+KO group, followed by HFD+WT, HD+KO and ND+WT mice. Data were representative images or were expressed as the mean ± SD of *n = 3* experiments. *, P < 0.05.
