# Supplementary figures and images for: ACE2 deficiency exacerbates obesity-related glomerulopathy through its role in regulating lipid metabolism
Source: Cell Death Discov. 2022 Sep 30;8:401. doi: 10.1038/s41420-022-01191-2 (PMC9523180; doi:10.1038/s41420-022-01191-2)

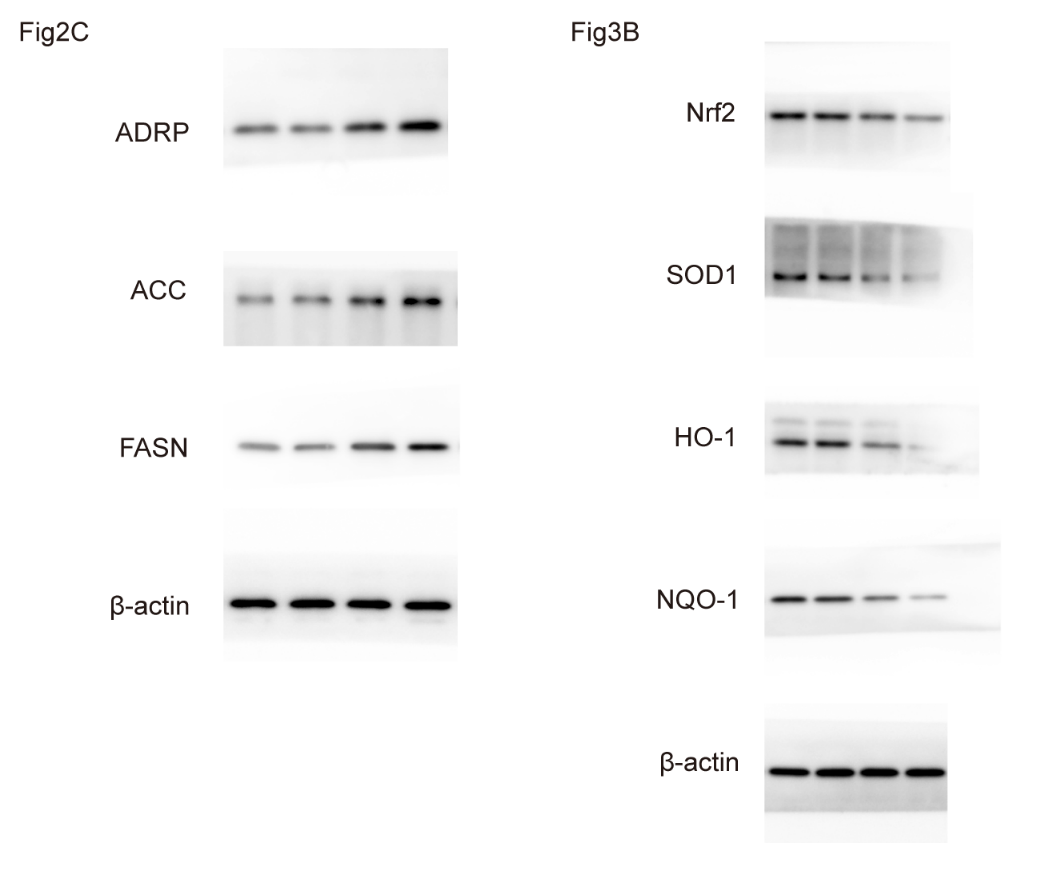


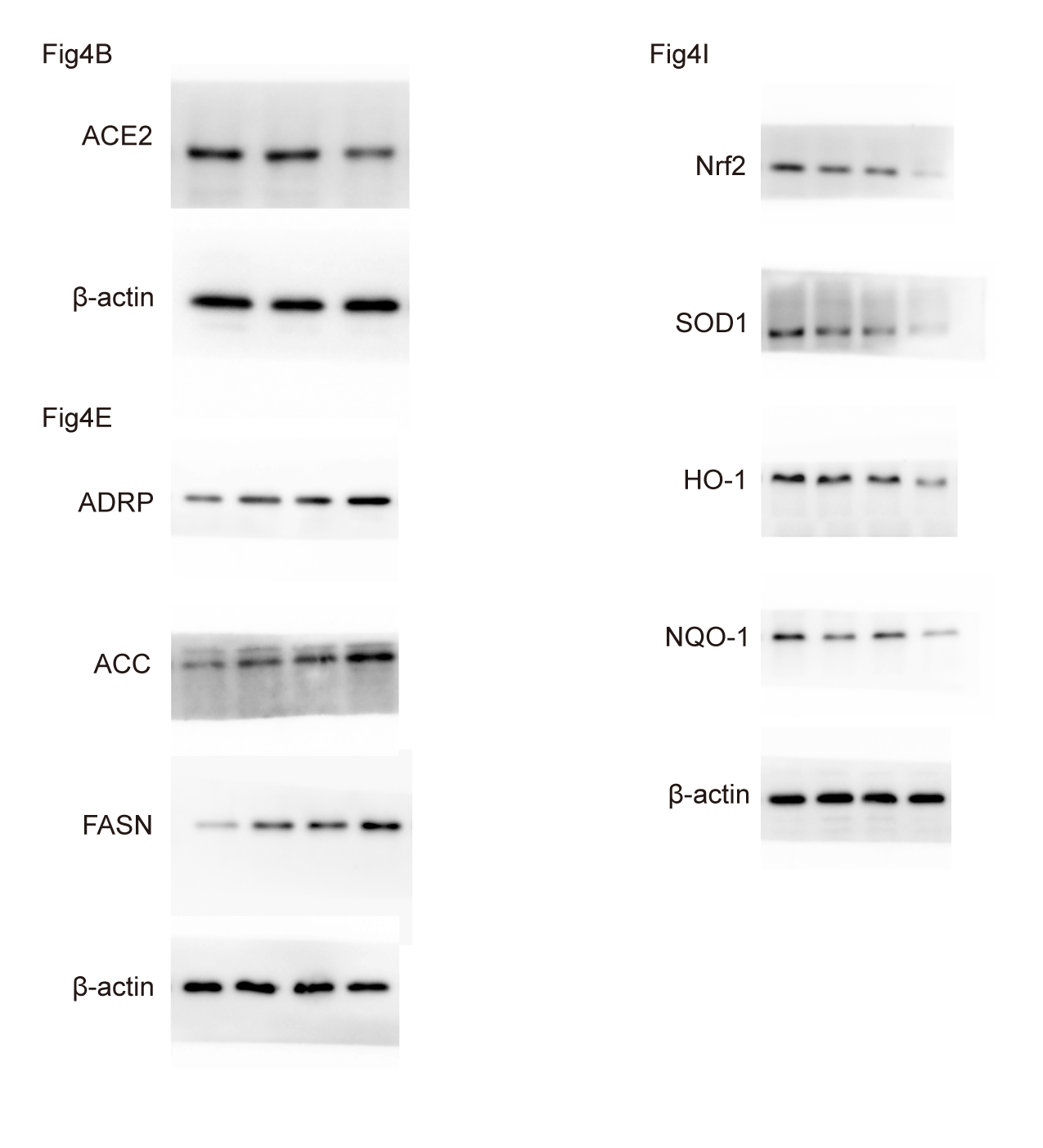

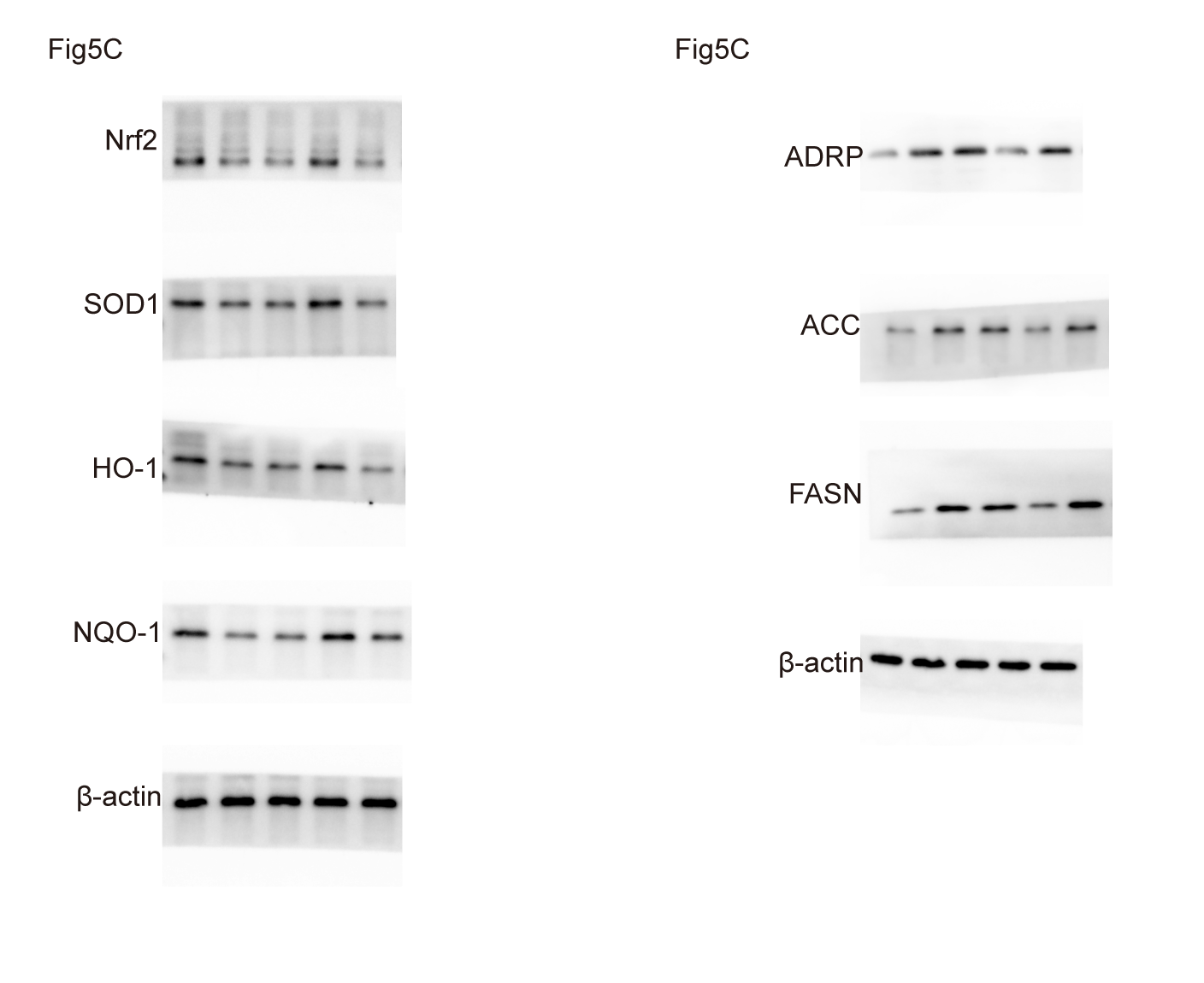

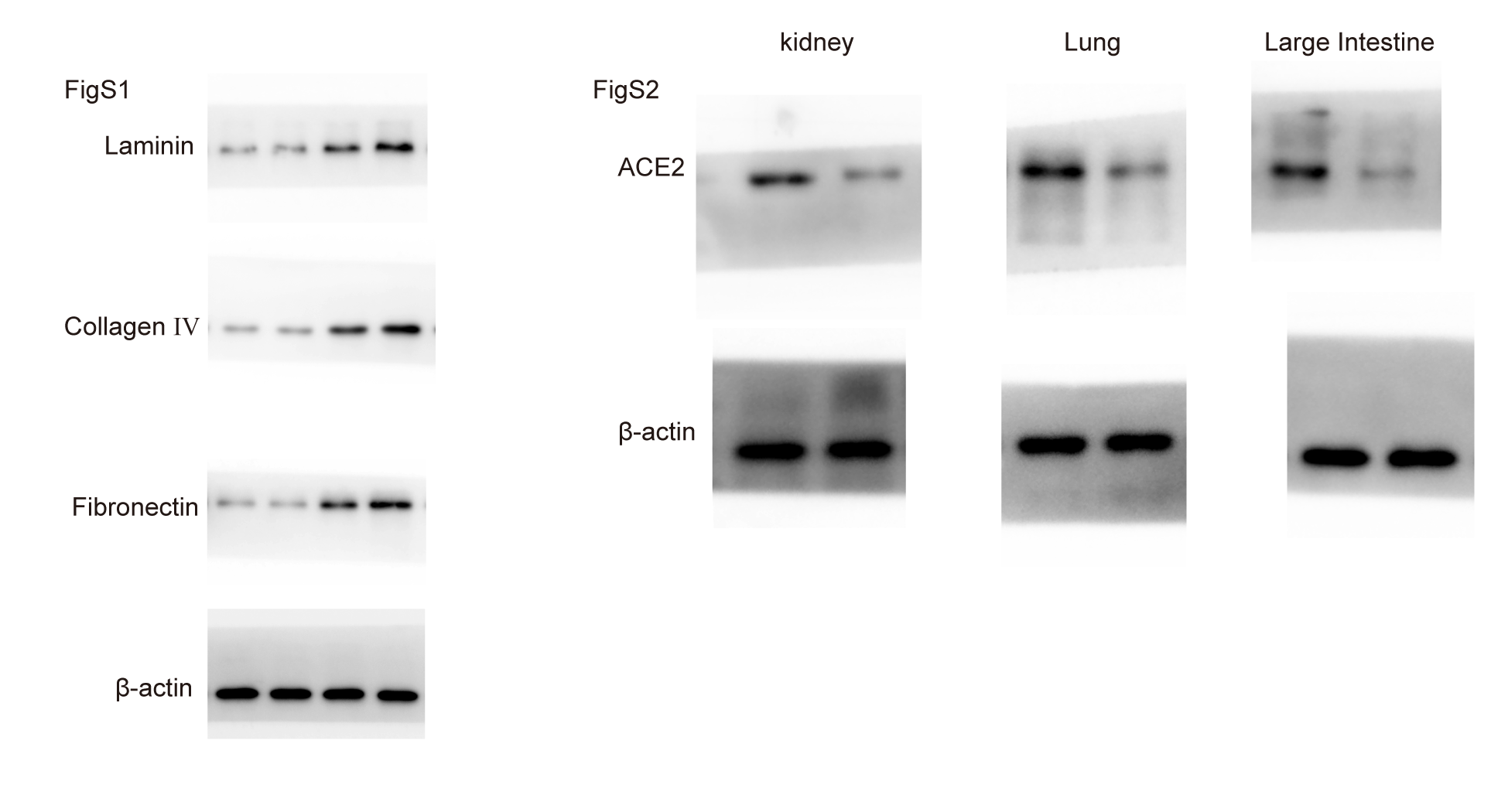

Supplement: Supplementary file 2 — Original Data File [file 41420_2022_1191_MOESM2_ESM.docx]

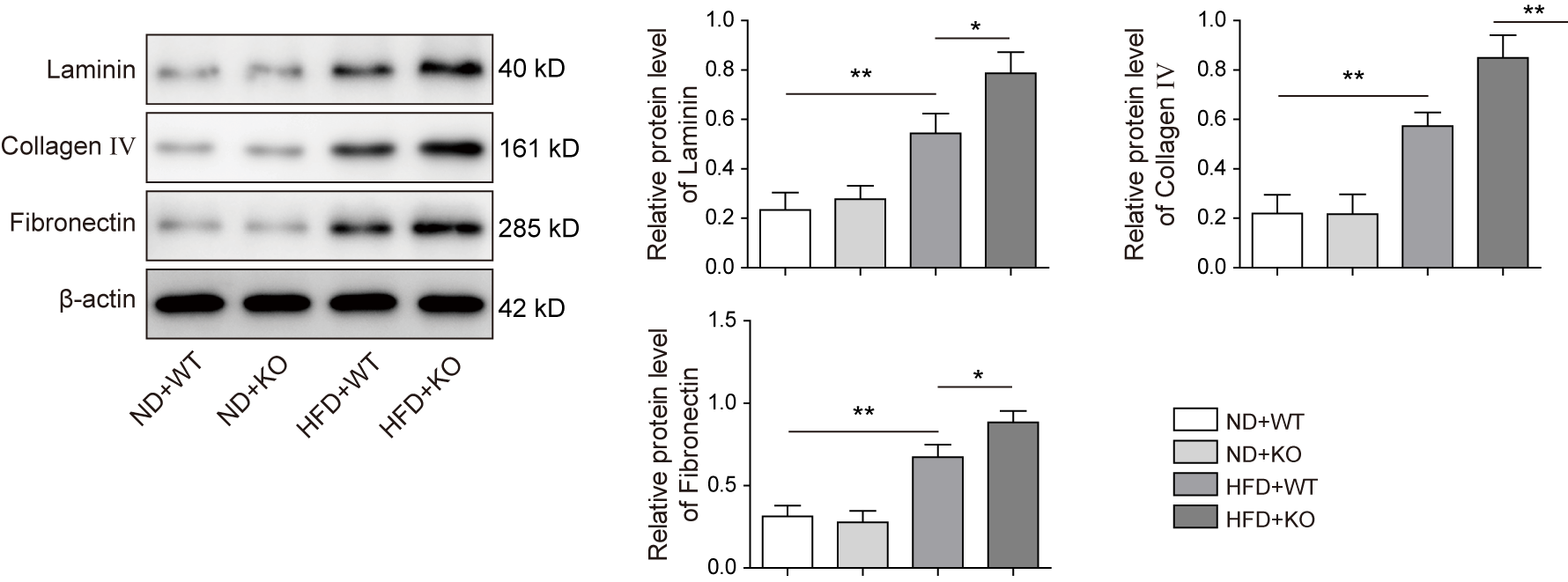

Supplement: Supplementary file 3 — Figure S1 [file 41420_2022_1191_MOESM3_ESM.tif]

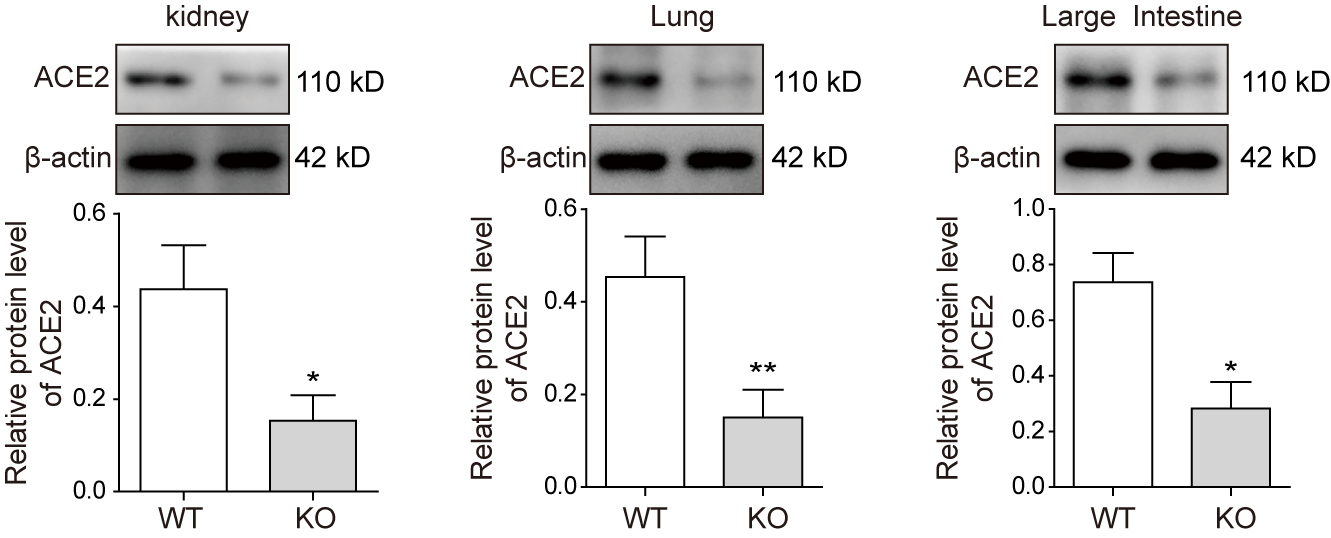

Supplement: Supplementary file 4 — Figure S2 [file 41420_2022_1191_MOESM4_ESM.tif]

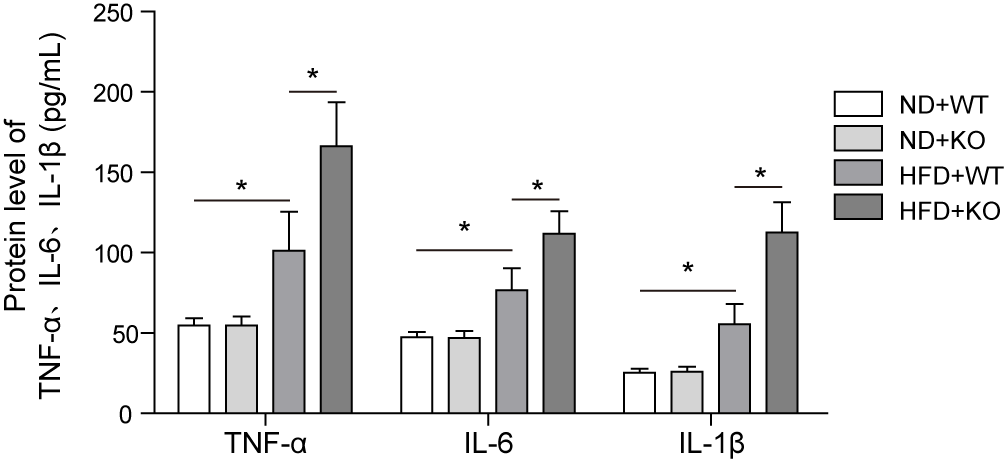

Supplement: Supplementary file 5 — Figure S3 [file 41420_2022_1191_MOESM5_ESM.tif]
